# Removal of Phenol by *Rhodococcus opacus* 1CP after Dormancy: Insight into Enzymes’ Induction, Specificity, and Cells Viability

**DOI:** 10.3390/microorganisms12030597

**Published:** 2024-03-16

**Authors:** Natalia S. Egozarian, Elena V. Emelyanova, Nataliya E. Suzina, Olesya I. Sazonova, Valentina N. Polivtseva, Tatiana O. Anokhina, Yonghong Wu, Inna P. Solyanikova

**Affiliations:** 1 G.K. Skryabin Institute of Biochemistry and Physiology of Microorganisms, Pushchino Scientific Center for Biological Research of the Russian Academy of Sciences, Prospect Nauki 5, Pushchino 142290, Russia; natae05@yandex.ru (N.S.E.); elenvem@ibpm.pushchino.ru (E.V.E.); suzina_nataliya@rambler.ru (N.E.S.); sazonova_oi@rambler.ru (O.I.S.); kaistia@gmail.com (V.N.P.);; 2 State Key Laboratory of Soil and Sustainable Agriculture, Institute of Soil Science, Chinese Academy of Sciences, 71 East Beijing Road, Nanjing 210008, China; yhwu@issas.ac.cn; 3 Regional Microbiological Center, Institute of Pharmacy, Chemistry and Biology, Belgorod National Research University, Pobeda Street, 85, Belgorod 308015, Russia

**Keywords:** *Rhodococcus opacus* strain 1CP, dormancy, degradation, phenol, immobilization, box-PCR

## Abstract

Biodegradation of phenol is an effective method for removing this toxicant from contaminated sites. Phenol is a toxic compound for living cells, so many bacteria degrade phenol in relatively low concentrations, up to 0.75 g L^−1^. The *Rhodococcus opacus* strain 1CP is an effective destructor of a wide range of pollutants. In the absence of a carbon source in the medium, cells of the *R. opacus* 1CP strain easily form cyst-like resting cells (CLC). The purpose of this work was to evaluate the viability of cells during long-term storage and the efficiency of the process of phenol destruction by *R. opacus* 1CP cells germinating after dormancy. Resting cells were obtained by simple cultivation in a rich medium followed by storage under static conditions. This is a simple approach to obtain a large amount of biomass. Decomposition of phenol proceeded via catechol followed by *ortho*-cleavage of aromatic ring. The induction of three phenol hydroxylases was detected by RT-PCR in cells germinated in a mineral medium with phenol as the carbon source. The stability of the genome of cells germinating after dormancy is shown by box-PCR. Dormant *R. opacus* 1CP cells, both suspended and immobilized, can be directly used for the decomposition of phenol after 4–12 months storage. In addition to phenol, after 9 months of storage, immobilized germinating cells easily metabolized 4-chlorophenol and 2,4,6-trichlorophenol. The results demonstrate a potential and simple approach toward achieving long-term storage of cells for further use in bioremediation.

## 1. Introduction

Phenol and its derivatives are byproducts of various industrial processes, including oil refineries, steel mills and blast furnaces. These compounds can be found in sewage waters of oil refineries, plastics plants, artificial resins, wood–chemical plants, plants of organic dyes, particle boards, non-ferrous metallurgy plants, and others [1,2,3,4]. The concentration of phenol in sewage can reach up to 0.5 g L^−1^ [1,5] and in some cases, up to 30 g L^−1^ (http://enviropark.ru/course/info.php?id=49, accessed on 17 November 2021). The human health noncancer phenol values for drinking and nondrinking water sources are 2.0 μg L^−1^ and 2.3 μg L^−1^, respectively (https://www.epa.gov/sites/production/files/2015-06/documents/in_hh_406_nc_07141999.pdf, accessed on 17 November 2021). Phenol can have negative effects on organisms due to its ability to separate oxidative phosphorylation, disrupt the integrity and function of bacterial cell membranes, and cause heat stress. Additionally, the intermediate of phenol degradation (catechol) causes oxidative stress in some bacterial species [6]. Phenol can prevent its own oxidation by inhibiting the activity of catechol 1,2-dioxygenase (1,2-CDO)—an enzyme responsible for cleavage of catechol, the first intermediate in the phenol degradation pathway.

There are many ways to carry out the chemical treatment of phenol-containing wastewater, with notable approaches including advanced oxidation technologies (heterogeneous and homogeneous photocatalysis, electrochemical oxidation, ultrasound radiation, wet air oxidation, ozonation, Fenton-like reactions and their combinations) [7], e.g., the heterogeneous photo-Fenton process [8]. Simultaneously, the employment of microbial remediation methods for the treatment of environmental organic contaminants, including phenol, has received widespread attention [9,10,11,12,13]. These techniques are often distinguished by their high efficiency, low cost, and environmental sustainability [14]. Fungal and bacterial approaches can be used either alone or combined with physicochemical methods [15]. Coupled systems between Fenton AOPs and white rot fungi for environmental organic pollutant remediation have demonstrated efficiency [16]. The most active bacterial strain metabolizing phenol and its derivatives were isolated from environments polluted with these compounds [17,18]. The usage of biological preparations based on individual or mixed bacterial cultures is often the most effective way to remove phenolic contamination. In general, bacteria are capable of degrading phenol in relatively low concentrations, up to 0.75 g L^−1^. For example, a strain of *Pseudomonas* sp. phDV1 decomposed phenol at concentrations up to 600 mg L^−1^ [19]. Strain *Rhodococcus* sp. SKC has been tested for its ability to degrade phenol with a *K*_i_ of 418.79 mg L^−1^ [20]. Among the most effective phenol destructors is a bacterium such as *Burkholderia* sp. BNS [21]. Immobilization protects bacterial cells from the negative effects of toxic compounds and increases the concentration of toxicants for biodegradation. Thus, alginate-immobilized *Pseudarthrobacter phenanthrenivorans* Sphe3 cells decomposed phenol at a concentration of up to 1500 mg L^−1^ [22]. Chitosan-immobilized bacterial cells *Chitinophaga* spp., *Methyloversatilis* spp., *Terrimonas* spp. and *Pseudomonas* spp. decomposed decabromodiphenyl ether, while also demonstrating stability under the conditions of long-term UVA irradiation and the presence of high-level free radicals [23]. The use of biological preparations is limited by a number of issues, such as maintaining cell viability, maintaining a metabolically active state, and the time required for the cells to resume active growth. Immobilization not only protects cells from stress, but also extends cellular lifespan. Polyvinyl alcohol, alginate and some other additives are suitable matrix for cell immobilization. Activated sludge applied to such a hybrid matrix survived at phenol concentrations of up to 2000 mg L^−1^, and the degradation efficiency increased from 15 to 34% [24]. Alginate-biopolymer-immobilized *Pseudomonas oleovorans* ICTN13 cells showed more than 20% increased efficiency in phenol degradation compared to free cells. Encapsulation of cells increased their life time to 30 days [25]. The positive effect of immobilization in polyvinyl alcohol–alginate–kaolin beads was also shown in the case of the strain *Sphingomonas* sp. GY2B [26]. The authors showed that the effect manifested itself primarily through reducing the degradation time and improving phenol degradation rate. However, the initial phenol concentration, 100 mg L^−1^, was low [26]. In addition to immobilization in gels, lyophilization is an effective approach for cell preservation. This method can significantly reduce the mass of the biopreparation. For example, studies have been carried out on the effect of the lyophilization stage on the viability of *Rhodococcus* cells and the possibility of their subsequent use in biotechnologies for cleaning environmental oil pollution [27]. The use of lyophilized cells made it possible to obtain 47% removal of total petroleum hydrocarbons after 15 days of the experiment. However, lyophilization is accompanied by cell death, and the preparation of an active cell mass requires special media and time. The use of biopreparations in climatic zones with a limited period of optimal temperatures requires the usage of highly active microorganisms with high-speed metabolism. 

However, there are some drawbacks to biological technologies, including a longer time required for degradation and the potential for certain toxicants to break down into more toxic by-products. Additionally, introducing enriched microbes to the contaminated soil can be challenging due to variations in environmental factors (temperature, pH, moisture content, nutrients) or competitive inhibition by other microbes. Finally, maintaining the vitality and metabolic activity of cells in biological preparations can be difficult, and it may take time for cells to resume active growth. Earlier, it was shown that cells of actinobacteria have the ability to enter a dormant state, characterized by the formation of cyst-like cells (CLC), under adverse conditions such as starvation, nitrogen limit, and drying [28,29]. When CLCs are re-introduced to a rich medium, they can resume growth quickly (less than 1 h). This is particularly evident for the strain *Rhodococcus opacus* 1CP, where a population of cells growing from such resting cells was able to effectively destroy toxicants that had not been metabolized by the culture or process, which may have taken a significant period of time [28]. Additionally, observations have shown that cells of actinobacteria, stored in a suspension of buffer for an extended period, can rapidly renew growth while maintaining their destructive potential [29]. It is worth noting that non-spore-forming bacteria can easily enter a dormant state, allowing survival in adverse conditions. However, when favorable conditions arise, these cells can quickly exit their dormant state and resume growth, with the ability to rapidly decompose toxic compounds. Despite this, the preservation of destructive activity and the rate at which germinating cells can decompose toxic substances remain poorly studied.

The aim of this research was to develop methods for obtaining dormant *R. opacus* 1CP cells, investigate their stability and germination characteristics, and utilize them for the remediation of phenol-contaminated wastewater.

## 2. Material and Methods

### 2.1. Chemicals

The reagents used to prepare the mineral medium were of high analytical purity grade (Reachim, Moscow, Russia). The biochemical reagents were obtained from various suppliers, including Sigma (St. Louis, MO, USA) and Serva (Heidelberg, Germany), while 3-methylcatechol was obtained from Koch-Light (Cambridge, UK) and 4-methylcatechol was obtained from Fluka (Buchs, Switzerland). Phenol was sourced from KhimMed, Moscow, Russia.

### 2.2. Bacterial Culture and Cultivation Conditions

The study focused on a Gram-positive, non-spore-forming bacterium named *Rhodococcus opacus* 1CP (DSM 46757 and VKM Ac-2638), which was isolated from enrichment medium containing 2,4-dichlorophenol [21]. The strain was cultivated in a mineral medium of the following composition (g L^−1^): Na_2_HPO_4_, 0.7; KH_2_PO_4_, 0.5; NH_4_NO_3_, 0.75; MgSO_4_ × 7H_2_O, 0.2; MnSO_4_, 0.001; FeSO_4_, 0.02 [30]. The inoculum was cultivated in 750-mL flasks (work volume 200 mL). A mineral medium contained phenol (0.2–3.0 g L^−1^ depending on experimental conditions described in the Result section) as the sole source of carbon and energy. The cells were grown and agitated under 220 rpm at 29 °C. The disappearance of the substrate was monitored by the absorption of the culture medium at the absorption peak of phenol (268 nm). In this experiment, an aliquot of the cell culture liquid was taken, and the cells were separated by centrifugation at 12,000× *g* for 3 min at room temperature (RT) (Eppendorf, Hamburg, Germany). The resulting supernatant was then recorded in the 240–340 nm wavelength range using a UV–Vis 1800 spectrophotometer (Shimadzu, Kyoto, Japan).

The colony-forming ability of cells (CFU mL^−1^) was determined during inoculations of cell suspensions diluted 10^N^ times on the plates with solid media (2.0% *w/v* of agar) and incubated at 29 °C for 5 days.

### 2.3. Obtaining Cell Preparations

Dormant cells were obtained by cultivation of *R. opacus* 1CP in tubes with 10 mL of Luria–Bertani (LB) medium on a stir plate with 220 rpm at a temperature of 29 °C for 3 days until the OD reached 1.3–1.4. Afterwards, the sterilized cultures were stored statically at room temperature for a period of 4 months. Test tubes with the grown culture (15 per pack) were placed in a plastic bag to prevent evaporation of liquid through a cotton plug and stored at room temperature. The volume of the culture medium, equal to 10 mL, allows for storage at room temperature for up to 1 year without complete evaporation of the liquid. Since all the cells in each tube were used for subsequent experiments, this allowed for an approximately consistent number of bacterial cells to be worked with. Before using the cells, they were washed with a sterile medium of the appropriate composition (LB or mineral one), resuspended, and prepared for further experiments.

### 2.4. Preparation of Dormant Cell for the Determination of Respiratory Activity

The sterile dormant cell suspensions contained in one tube were centrifuged at a high speed of 12,000× *g* for 3 min at room temperature (RT). The cells were then resuspended in 15 mL of 50 mM Tris–HCl solution (pH 8.0). Next, the cells were aerated by stirring for a set period of time, which was 30 min, 1, 2, 4, 6 and 8 h. After each aeration time, 1 mL of the cell suspension of the culture was used for measuring the respiration activity and ability to remove benzoate or (chlorinated) phenols. Analysis of respiratory activity is described in Section 2.11.

### 2.5. Preparation of Germinating Cells for the Determination of Enzymatic Activity

The sterile cell suspensions were centrifuged as previously described and the supernatant was discarded. The cells were then resuspended in 100 mL of mineral medium and 200 mg L^−1^ of a specific substrate (benzoate, phenol) was added with stirring. The growth of the culture was monitored by measuring the increase in optical density at OD_545_ nm and the decrease in substrate concentration over time, as described above.

### 2.6. Preparation of Cell-Free Extracts

The cells were destroyed using a Hughes-type press (IBPM-press, Pushchino, Russia) that applies extremely high pressure 3200 kg/cm^2^ to cause disintegration. After the cells were broken up, the cell debris was removed by centrifugation at 10,000× *g* (4 °C, 30 min) in the presence of trace amounts of DNAase. The resulting supernatant was then used to determine the enzyme activity.

### 2.7. Determination of Enzyme Activity and Protein Amount

The activity of various enzymes was determined using spectrophotometry UV-1800 (Shimadzu, Japan) at 25 °C. The activity of catechol 1,2-dioxygenase, catechol 2,3-dioxygenase and gentisate 1,2-dioxygenase was determined by the rate of *cis*,*cis*-muconate (λ = 260 nm, ε = 16,900 M^−1^ cm^−1^), 2-hydroxymuconic semi-aldehyde (λ = 375 nm, ε = 33,400 M^−1^ cm^−1^), and maleyl pyruvate (λ = 330 nm, ε = 10,800 M^−1^ cm^−1^) formation in the reaction mixture, respectively [31,32]. The activity of protocatechuate 3,4-dioxygenase was determined by a reduction of protocatechuate in the reaction mixture (λ = 290 nm, ε = 2870 M^−1^ cm^−1^) [33]. However, the activity of protocatechuate 4,5-dioxygenase was determined by the formation of 2-hydroxy-4-carboxymuconic semi-aldehyde at λ = 410 nm (ε = 11,200 M^−1^ cm^−1^) [34]. The activity of protocatechuate 2,3-dioxygenase was examined by measuring the rate of substrate-dependent oxygen consumption. A 2-mL assay mixture contained 50 mM GTA buffer (pH 7.3) consisting of 50 mM 3,3-dimethylglutarate, 50 mM Tris, 50 mM 2-amino-2-methyl-1,3-propanediol, crude extract, and 100 mM protocatechuate (PCA). The reaction mixture was incubated at 35 °C, and the oxygen consumption rate was determined with an oxygen electrode (Oakton^®^ DO ^6+^ Dissolved Oxygen Meters, Cole-Parmer, Vernon Hills, IL, USA) [35]. The activities of phenol hydroxylase and salicylate hydroxylase were determined by the rate of NADH uptake at λ = 340 nm in the presence of 0.1 mM FAD and corresponding substrate. However, the consumption of NADH by the cell-free extract in the absence of phenol/salicylate was considered as control. The activity of muconate cycloisomerase (MCI) was determined by recording substrate depletion at λ = 260 nm (ε = 16,900 M^−1^ cm^−1^) [36]. The specific activity of the enzymes was expressed in micromoles of the substrate used or the resulting product for 1 min per 1 mg of cellular protein. The protein concentration was determined using spectrophotometry with the modified Bradford method [37]. A unit of activity was defined as the amount of enzyme catalyzing the conversion of 1 µmol of substrate or the formation of 1 µmol of product per minute. Relative activity was calculated as 100% activity with unsubstituted or better substrate.

### 2.8. RNA Isolation and cDNA Synthesis

Cells were disrupted by suspension in ExtractRNA reagent (Evrogen, Moscow, Russia) and vortexing with glass beads (Sigma, St. Louis, MO, USA) for 2 min. RNA was isolated following the manufacturer’s instruction, treated with RNA free DNAse I (Thermo Scientific, Waltham, MA, USA) and purified using a GeneJET RNA purification kit (Thermo Scientific, USA). cDNA was synthesized with a cDNA Synthesis kit (SybEnzyme, Novosibirsk, Russia) as per the manufacturer’s recommendations.

### 2.9. Real-Time PCR

Real-Time PCR was performed using a LightCycler 96 system (Roche, Indianapolis, IN, USA) with specified parameters: 95 °C for 5 min; 45 cycles: 95 °C for 30 s, 60 °C for 20 s, 72 °C for 30 s. RNA polymerase *beta* subunit and 16S RNA reference genes were used in Real-Time PCR for cDNA samples from dormant, phenol-grown, and benzoate-grown cells, each analyzed in three biological and two technical replicates. Gene expression levels were calculated following the manufacturer’s instructions, represented by averages based on RNA polymerase or 16S RNA reference genes of the same strain. Gene activation was estimated by ratios of benzoate cells/dormant cells and phenol cells/dormant cells. Primer sequences for Real-Time PCR are listed in Table 1.

### 2.10. REP-PCR

To differentiate genotypic variations among the samples, genomic fingerprints were conducted using primers A1R and (GTG)5 [38], following Mohapatra et al.’s amplification program [39]. The PCR was performed in a GeneAmp PCR System 9700 by Applied Biosystems, Waltham, MA, USA, in a 25 μL reaction mixture. The PCR mix included 100 ng DNA template, 2 µM primer, 1 × PCR buffer, 200 µM deoxyribonucleoside triphosphates, 3 mM MgCl_2_, 5% DMSO, 0.1 mg mL^−1^ bovine serum albumin, and 2.5 units of DreamTaq polymerase. Box A1R sequence: 5′-CTACGGCAAGGCGACGCTGACG-3′. The amount of template DNA in the rep-PCR reaction was the same. Samples were applied with a buffer of the following composition: 0.025% xylene cyanol, 0.025% bromophenol blue, and 2.5% ficol (type 400). The GeneRuler 1 kb Plus DNA Ladder standard from ThermoScientific (Vilnius, Lithuania) was used as a marker DNA. Electrophoresis was carried out in a 1.2% agarose gel and 0.5× Tris-borate buffer as standard procedure [40], with DNA visualization using ethidium bromide staining. The gel images were captured using a BioTestColor system v.2.2 (KekLab, Moscow, Russia).

### 2.11. Polarographic Determination of Respiratory Activity, Benzoate 1,2-Dioxygenase (BDO) and Phenol Hydroxylase (PH) Activity 

Measurements were performed in air-saturated 50 mM Tris˗HCl buffer (pH 8.0) at room temperature in an open 5-mL cuvette with a stirrer. When a basal level of cell respiration was stabilized, the determination of respiratory activity, BDO and PH activity were carried out. 1CP cells contained endogenous substrates. Cellular respiration due to endogenous substrates was determined as basal respiration.

For the determination of respiratory activity of 1CP cells (cellular respiration in the absence of exogenous substrate), stirring was stopped after stabilization of basal level of cells’ respiration. Oxygen concentration change rate was measured using a Clark-type oxygen electrode. The rate was recorded with a two-coordinate XY Recorder-4103 (manufacturer, city, Laboratorni Přistroje, Praha, Czech Republic). The rate of respiration was measured in μg O_2_ (L s)^−1^.

Enzyme activity (BDO or PH) was evaluated by observing the change of 1CP cells’ respiration in the presence of enzyme substrate. Substrates such as phenol, substituted phenols, benzoate, etc., were injected into the cuvette for the determination of enzyme activity and oxygen concentration change rate was measured with the Clark-type oxygen electrode. The Clark-type oxygen electrode transduced the chemical signal (the change of oxygen concentration) into the electrical signal (the change of the electrode current). The oxygen electrode was equipped with an Ingold 531 O2 Amplifier of signal (Switzerland-USA). The signal was recorded with a two-coordinate XY Recorder-4103 (Czech Republic). The recorded signal reflected the rate of the enzymatic reaction of BDO or PH with substrate. The unit of rate was pA s^−1^ (1 pA s^−1^~0.153 μg O_2_ (L s)^−1^).

### 2.12. Cells Immobilization

The suspension of dormant cells (OD_545_ 0.648) was utilized to immobilize them on polycaproamide fiber. A 20 mL cell suspension was added to 2.0 g fiber in 200 mL total volume of cultivation medium. Immobilization was performed for 17 h with agitation. Later, 100 mg L^−^^1^ phenol was added to each flask and induction of cells was performed during another 24 h. Non-immobilized cells were washed out with a sterile medium and examined under a microscopy. The supernatant was tested for the presence of phenol. Fresh mineral medium (200 mL) was added to fiber with immobilized cells. Phenol was added at various concentrations (0.25, 0.5, 1, 2, 3 g L^−^^1^) for testing. Microscopy was used to study the immobilized cells after 5 and 10 days. The carrier with 200 mL medium and 1 g L^−^^1^ phenol served as the chemical control.

### 2.13. Microscopic Techniques

#### 2.13.1. Phase Contrast Microscopy

Phase contrast microscopy was performed using a Nikon Eclipse Ci microscope (Nikon, Tokyo, Japan) equipped with a Jenoptic ProgRes^®^ SpeedXTcore5 camera (Jenoptik, Jena, Germany) and Axioplan (Carl Zeiss, Oberkochen, Germany).

#### 2.13.2. Scanning Electron Microscopy

The surface morphology of the biofilms on the polycaproamide fiber was examined using scanning electron microscopy (SEM). Samples of the cells placed on membrane filters were fixed in glutaraldehyde vapor for 24 h at 4 °C and post fixed in OsO_4_ vapor for 3 h at 20 °C. After dehydration sequentially in ethanol and tert-Butanol (Sigma-Aldrich, USA), the samples were dried in the JFD-320 Freeze Drying Device (JEOL, Tokyo, Japan), coated with gold dust in JFC-1600 auto fine coater (JEOL, Tokyo, Japan), and then placed on SEM stubs for examination under a JSM-6510LV SEM (JEOL, Tokyo, Japan).

## 3. Results

### 3.1. The Ability of R. opacus 1CP to Form Dormant Cells

The growth of *R. opacus* 1CP in LB medium resulted in an increase in the optical density (OD) of the culture. CFU per 1 mL after 3 days of cultivation was 10^12^. CFU remained stable for the first month, then decreased in CFU by two orders of magnitude due to prolonged storage. Dormant cells formed after 4 months of starvation in steady conditions. The determination of storage duration was based on literature and previous observations of *R. opacus* 1CP behavior. The dormant state was confirmed by cytological features, inactive respiration, and resistance to damage [41].

**Figure 1:**
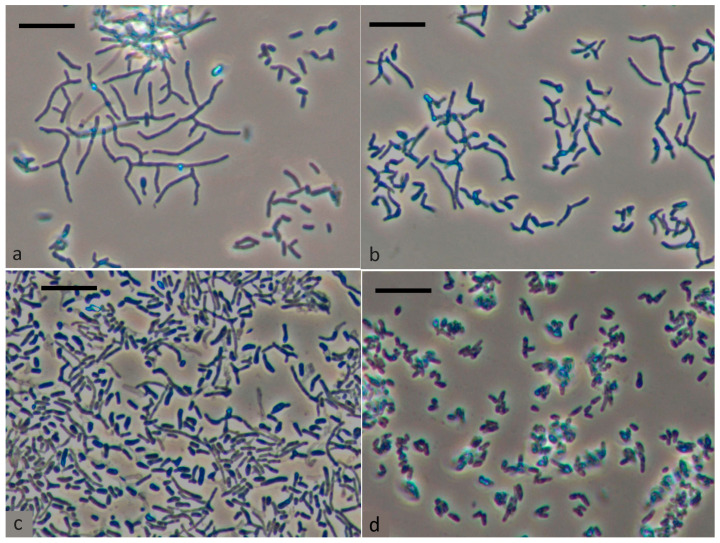
Morphological changes of *R. opacus* 1CP cell during incubation in liquid LB medium for 1 day (**a**), 2 days (**b**), 5 days (**c**), and after long-term storage for 4 months (**d**). The scale bar represents 10 μm.

Figure 1 presents changes in the morphology of vegetative cells grown in a rich environment during storage. Rhodococci are known for transitioning between rod-shaped and coccoid forms, with the morphogenetic cycle starting with coccus or short rod states. The next generation of cocci or short rods is formed by fragmentation of rods, filaments and hyphae. Development of branched rods is observed when growing on a rich medium. When growing on a rich medium, as can be seen from the data presented, the development of branched rods is observed (Figure 1a–c). After 4 months of storage, cells showed rounded shape, thickened cell wall, outer capsular layer, large electron-transparent inclusions in cytoplasm, and electron-dense homogenous cytoplasm. Respiratory activity showed no bright-red fluorescence typical of metabolically active dividing cells in direct microscopic tests with CTC tetrazolium dye [28]. Microscopic examination of culture samples stored at room temperature showed that up to 80% of cells were large and rounded after 4 months of storage (Figure 1d). Moreover, the percentage of such cells in the preparation was significantly higher than what was previously shown.

**Figure 2:**
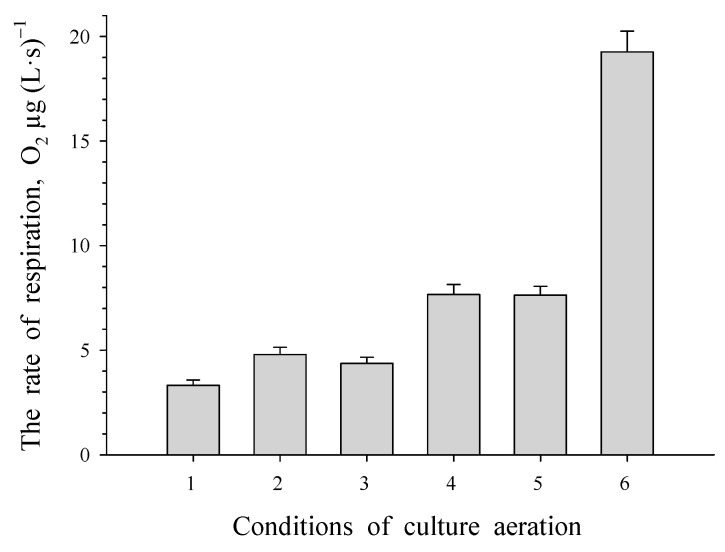
Respiratory activity of dormant *R. opacus* 1CP cells at different time intervals of aeration: 0.5, 2, 4, 6, and 26 h (labeled as 1, 2, 3, 4 and 5, respectively) as well as after 20 h of aeration with benzoate present (labeled as 6).

The study of the level of respiratory activity in resting cells (Figure 2) revealed an initial increase from 3 μg O_2_ (L s)^−1^ to 8 μg of O_2_ (L s)^−1^ after 30 min and 6 h of aeration, respectively. This oxygen consumption level was maintained at 8 μg of O_2_ (L s)^−1^ for up to 26 h of aeration. Dormant cells showed no activity with phenol, benzoate or their substituted analogues, but adding benzoate resulted in a rise in oxygen consumption to 20 μg of O_2_ (L s)^−1^ in aerated dormant cells.

### 3.2. REP-PCR

PCR fingerprinting was used to detect potential genomic alterations in *R. opacus* 1CP when utilizing phenol as the sole carbon and energy source. We examined DNA samples from *R. opacus* 1CP cells that had been subcultured for an extended period in two independent laboratories (cells did not undergo a dormant stage) and compared them to a DNA sample from cells grown in a phenol-containing mineral medium after a resting period. The analysis of the obtained results showed that primer (GTG)5 is more informative.

**Figure 3:**
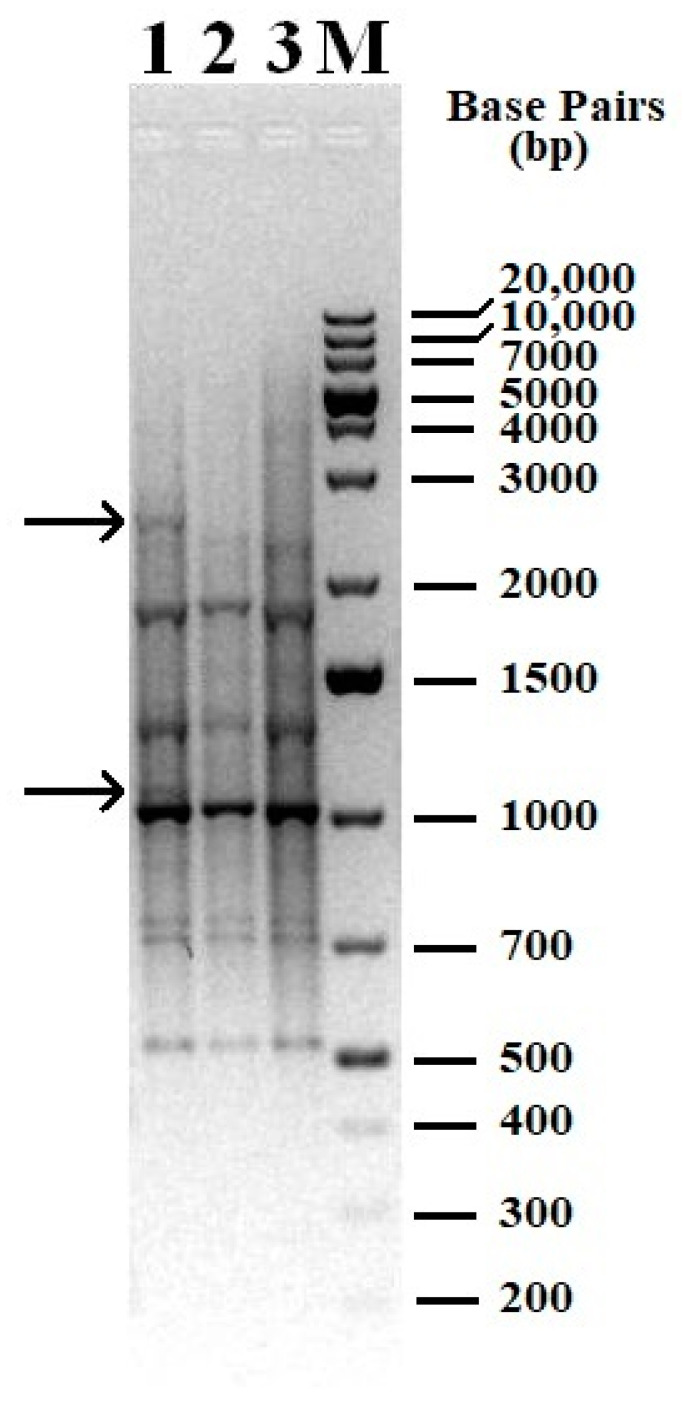
Genomic fingerprint of the studied samples with (GTG)_5_ primer. 1—DNA from dormant cells was used as a matrix; 2—DNA isolated from cells of a bacterial culture subcultured in laboratory 1 was used as a matrix; 3—DNA isolated from cells of a bacterial culture subcultured in laboratory 2 was used as a matrix; M—molecular weight marker, 1 kb Plus DNA Ladder. Arrows indicate bands (patterns) present only in the sample 1 (cells after dormancy).

REP-PCR followed by electrophoresis demonstrated in Figure 3 showed that the genomic fingerprint profiles of the samples which did not undergo a dormant stage shared similarities and differing slightly from the experimental samples. The findings suggest that when cultivated using phenol as the sole carbon and energy source, some genetic modifications occur in the *R. opacus* 1CP strain.

### 3.3. The Aromatic Ring-Cleavage Oxygenase Activities in Induced Germinating Cells

**Figure 4:**
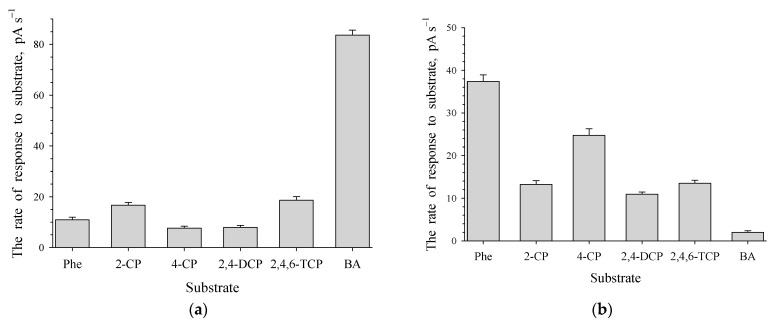
Responses to several aromatic compounds for *R. opacus* 1CP cells grown in the medium contained 0.2 g L^−1^ of benzoate (**a**) or 0.1 g L^−1^ of phenol (**b**) after the rest stage. Substrates and substrate analogues include: Phe—phenol; 2-CP—2-chlorophenol; 4-CP—4-chlorophenol; 2,4-DCP—2,4-dichlorophenol; 2,4,6-TCP—2,4,6-trichlorophenol; BA—benzoate.

Induction of dormant cells with benzoate (0.2 g L^−1^) or phenol (0.1 g L^−1^) resulted in a rapid increase in the OD of the culture liquid. Substrates were completely decomposed in less than 1 day. The activities of oxygen-dependent enzymes, phenol hydroxylase (PH) and benzoate 1,2-dioxygenase (BDO), of 1CP cells grown in a mineral medium with phenol or benzoate were determined. *R. opacus* 1CP cells grown with benzoate showed high activity with benzoate, but lower activity with phenol and mono- and dichlorophenols (Figure 4a). Cells grown with phenol showed high activity with unsubstituted phenols (Figure 4b). PH in whole cells was active with phenols, including chlorophenols. Notably, activity with certain monochlorophenols (2-CP and 4-CP), 2,4-DCP and 2,4,6-TCP was high, but lower than with phenol. Cells grown with phenol also showed low activity with benzoate.

### 3.4. RT-PCR Analysis of Cells Grown on Different Substrates

RT-PCR analysis was performed on RNA from actively growing cells to identify induced enzymes in cells germinating after dormancy. Table 2 presents data on gene activation for benzoate and phenol decomposition obtained by RT-PCR analysis. In the control, RNA was isolated from dormant cells. Activation of the BDO gene was significantly higher in cells germinating with benzoate. Only one out of four 1,2-CDO genes was activated. Increased transcription levels were observed for PCA 3,4-DO and phenol hydroxylases genes. Analysis of 1CP cells transcripts germinating with phenol showed a notable increase in one of 1,2-CDO gene transcription. PH3 transcription levels increased by nearly 2000 times among the three phenol hydroxylases.

### 3.5. The Activity of Enzymes in Phenol-Induced R. opacus 1CP Cell-Free Extract

The potential involvement of mono- and dioxygenases in decomposing phenol by this strain was tested in a cell-free extract from phenol-grown culture. The results of determination on specific enzymatic activity are presented in Table 3. The activity of extradiol dioxygenases, catechol 2,3-dioxygenase, protocatechuate 2,3- and 4,5-dioxygenase (PCA 2,3-DO, PCA 4,5-DO), was completely absent. Gentisate 1,2-dioxygenase (GDO) had no activity. Monooxygenases like phenol hydroxylase (PH), salicylate hydroxylase (SH) and *para*-hydroxybenzoate hydroxylase (PHBH) were also inactive. The activity of 1,2-CDO and PCA 3,4-DO are consistent with polarographic analysis results, both enzymes being active in the cell-free extract.

### 3.6. The Decomposition of Phenol by Cells Immobilized on a Fiber

**Figure 5:**
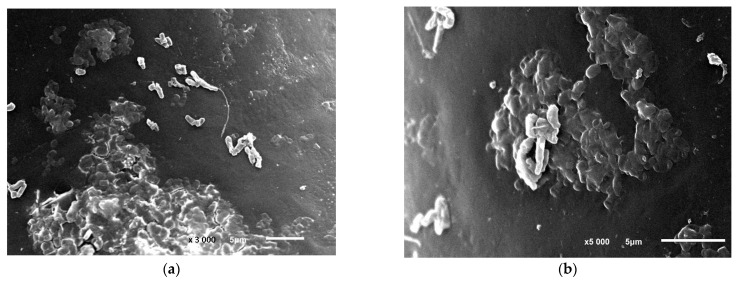
*R. opacus* 1CP cells during the colonization of the polycaproamide fiber in a mineral medium supplemented with phenol, captured through electronic photography. Two different areas of the polycarbonate fiber surface colonization: (**a**) magnification of 3000, bar—5 μm, (**b**) magnification of 5000, bar—5 μm. Electronic photography.

Direct immobilization of resting cells on the fiber for 17 h resulted in their complete sorption. In this case, the OD of the culture liquid in which cell sorption occurred was decreased from 0.261 (the beginning of immobilization) to 0.06 to 17 h. When 100 mg L^−1^ phenol was added to immobilized non-induced cells, it disappeared completely within 24 h. As immobilized cells were cultivated on phenol in a concentration of 0.25 to 3 g L^−1^, there was a gradual decrease of the toxicant in variants 0.25–1.0 g L^−1^ of phenol. The complete decomposition of 1.0 g L^−1^ of phenol was completed in 7.5 days. Immobilized cells were unable to degrade 1.5–3 g L^−1^ phenol without adaptation. The microscopic analysis (Figure 5) revealed that cells had colonized the polycaproamide fiber. This colonization led to a decrease in the time required for 0.25–0.5 g L^−1^ phenol destruction from 3.5 days to 1 day. Over a period of 2 months, the fiber became covered with cells. Cells immobilized on the fiber were active throughout the two months of the experiment, while monitoring was carried out. A gradual increase in the OD of the culture liquid was also monitored during the experiment.

**Figure 6:**
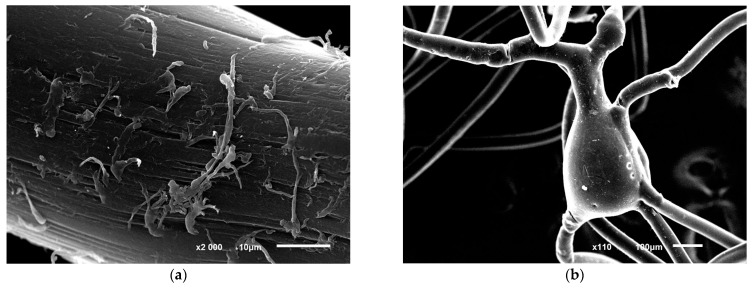

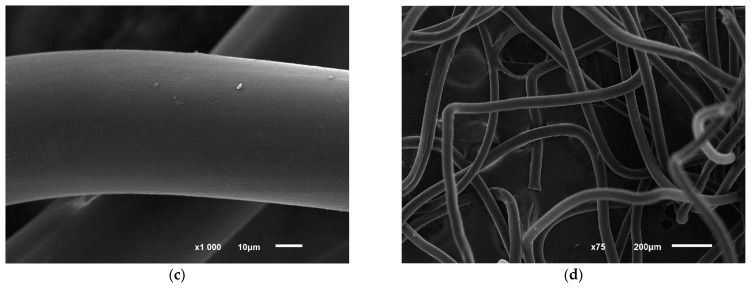
Photo of structural disorders in polycaproamide fiber with *R. opacus* 1CP cells immobilized, after undergoing 10 cycles of phenol decomposition (100 mg L^−1^), followed by storage for 5 months and 5 more phenol degradation cycles (**a**,**b**). The control image (**c**,**d**) shows the fiber in a mineral medium with phenol but without cells. Electronic photography.

Prolonged cultivation of immobilized cells in a mineral medium containing phenol led to a decrease in destruction. The inhibition was caused by a rise in end product levels, culture aging, and a shift in pH alongside phenol destruction. Replacing the previous growth medium with a fresh one triggered the resumption of phenol destruction. Extended cultivation of immobilized cells resulted in damage of the fiber. This was manifested in the violation of the fiber integrity, swellings, and other visible changes (Figure 6). It should be noted that under the conditions of our experiments, the absence of phenol sorption or degradation by the polycaproamide fiber was checked. In the control variant, mineral medium and polycaproamide fiber, the concentration of phenol added in an amount of 100 mg L^−1^ was not changed during the entire experiment and was detected in samples as described in Section 2.2. This indicated that a decrease in phenol concentration in the culture broth occurred due to biodegradation by the studied strain *R. opacus* 1CP.

### 3.7. Repeated Usage of Immobilized R. opacus 1CP Cells after Storage

Polycaproamide fiber with immobilized cells were repeatedly used after 5, 9 and 12 months of storage at 4 °C. Microscopic assay revealed that cells desorbed from the fiber after 5 months storage were presented by weak bridging cells, 7–10 µm long, with some parts appearing as CLC. Cell’s viability after storage was confirmed by the results of phenol destruction. Immobilized cells stored for 5 months did not lose their ability to degrade phenol. Initial 50 mg L^−1^ of phenol disappeared within 24 h, followed by consecutive additions of 100 mg L^−1^ every 24 h resulting in complete degradation. These results show high stability of immobilized cells. These immobilized cells can degrade 4-chlorophenol and 2,4,6-TCP. Complete destruction of initial 50 mg L^−1^ of 4-CP and 2,4,6-TCP took 2 and 4 days, respectively. Subsequent additions of these toxicants were degraded in 1 and 2 days, respectively. The efficiency of destroying phenol was not affected by storing the fiber with immobilized cells for 4 or 7 months. The addition of 50 mg L^−1^ of phenol to the storage medium led to substrate loss within 1 to 1.5 days of cultivation at 29 °C. Immobilized cells successfully decomposed 100 mg L^−1^ of phenol in 24 h after induction, showing the ability to destroy phenol for at least a year. In the control variant (fiber with phenol, without immobilized cells), no decrease in phenol was observed. Moreover, long-term use of immobilized cells with periodical substrate supply maintains their metabolic activity without loss. Storage resulted in minor changes in the fiber structure (Figure 6c,d).

## 4. Discussion

### 4.1. The Ability of R. opacus 1CP to Form Dormant Cells and Survive

In this study, we confirmed previous data presented on the experimental production of resting forms of actinobacteria *R. opacus* 1CP [28]. In contrast to the previously developed experimental approaches, a simple method was used to store cells by cultivating them in a rich medium until reaching maximum density, followed by static storage. The time of biomass storage was not limited. Maintaining a high CFU index with this method shows it is a simple and reliable method to obtain the biomass. Microscopy data revealed changes in cell morphology during storage—branching mycelium disintegrated, cells became shorter and thicker, raising questions about the duration of storage without affecting germination ability.

### 4.2. The Metabolic Capacity of Germinating Cells

Lack of metabolic activity is one of resting cells’ features. Resting cells, suspended in a buffer solution, showed low respiratory activity after 20 min of aeration (Figure 2), with no significant increase observed even with prolonged aeration. This limited respiration is attributed to the absence of growth substrates and possibly a lack of storage compounds in the resting cell. The cells exhibited no reaction to growth substrates such as phenol, benzoate, and chlorophenols. Induction of resting cells with phenol and benzoate led to the synthesis of enzymes with different substrate specificity, particularly related to the initial attack enzymes. In cells induced by phenol, the enzyme activity is highest against phenol, with phenol hydroxylase showing activity not only with phenol but also with monochlorophenols, 2,4-DCP and 2,4,6-TCP. When cells were exposed to phenol, they exhibited low activity against benzoate. Previously obtained data showed that strain *R. opacus* 1CP cells, grown on benzoate, had high benzoate activity and minimal activity with phenol and monochlorophenols [42,43]. Germinating cells aerated for 24 h with benzoate showed respiratory activity with monochlorobenzoate, unlike vegetative cells. This procedure may serve as a simple method to increase metabolic versatility of germinating cells. High respiratory activity was recorded with the addition of catechol and protocatechuate (PCA). Aerobic decomposition of phenol typically results in catechol formation in most cases [44]. An *Arthrobacter* strain degraded (0.5 g L^−1^) phenol in 65 h [45]. The induction of five enzymes from the 3-oxoadipate pathway and tricarboxylic acid cycle was confirmed by proteomic analysis [45]. Catechol is then cleaved in *ortho*- or *meta*-position to determines its degradation pathway [46]. Therefore, cells of *R. opacus* 1CP grown with phenol expected increased activity of the common enzymes of phenol degradation—PH, catechol 1,2-dioxygenase (1,2-CDO), muconate cycloisomerase. The lack of activity of extradiol-cleaving dioxygenases—catechol 2,3-dioxygenase, protocatechuate 2,3- and 4,5-dioxygenase (PCA 2,3-DO, PCA 4,5-DO), gentisate 1,2-dioxygenase (GDO), salicylate hydroxylase (SH) and *para*-hydroxybenzoate hydroxylase (PHBH) was expected. The increase in O_2_ consumption by cells in response to catechol addition is explained by the formation of this compound as a key intermediate in the decomposition of phenol by cells of this strain and by the induction of 1,2-CDO as a key enzyme responsible for *ortho*-cleavage of the resulting catechol. The high activity of protocatechuate 3,4-dioxygenase (PCA 3,4-DO) detected in the whole cells, is unusual for cells grown on phenol. However, the presence of PCA 3,4-DO is confirmed by the determination of the activity of this enzyme in the cell-free extract. Our comparative experiments on the activity of cells germinating after rest in LB media, in mineral medium with benzoate and with phenol showed significant differences in enzymatic activity patterns. The general pattern is that cells induced after the dormant stage were characterized by a wider substrate specificity compared to the variant in which cells actively grew in a rich medium. The data on 1,2-CDO and PCA 3,4-DO activity matched the polarographic analysis data. The activity of both enzymes was determined in the cell-free extract of *R. opacus* 1CP. In cell-free extract of *Halomonas organivorans* grown with benzoic acid (5 mM), cinnamic acid, (4 mM), salicylic acid (3 mM), phenylpropionic acid (4 mM), phenol (2.5 mM) and *para*-aminosalicylic acid (3 mM), catechol 1,2-dioxygenase activity was observed [47]. However, no activity towards protocatechuate was obtained [47]. To confirm the functionality of the characterized *catA* gene from *H. organivorans* coding for the 1,2-CDO, a specific enzymatic assay was performed. The activity of 1,2-CDO was measured in cell-free extract from *E. coli* H7 clone cells grown in the minimal medium supplemented with glucose, phenol and benzoic acid. 1,2-CDO activity was induced with phenol and benzoic acid, although a lower activity was detected in cell-free extract from cells grown with benzoic acid. In contrast, the activity of 1,2-CDO was not detected when glucose was added to the minimal medium [48]. Genes encoding the aromatic dioxygenase enzymes 1,2-CDO and PCA 3,4-DO were determined in three halophilic bacteria isolated from different saline environments and identified as *Halomonas organivorans*, *Arhodomonas aquaeolei* and *Modicisalibacter tunisiensis* growing on phenol in hypersaline media [2].

Data analysis using RT-PCR method showed a significant increase in gene activation related to enzyme decomposition of aromatic compounds in cells germinating after dormancy in the presence of benzoate. Activation of BDO gene was 3 orders of magnitude higher, while only 1 out of 4 genes encoding different types of 1,2-CDOes showed activation. Additionally, transcription levels of PCA 3,4-DO gene increased, corresponding to enzyme activity in cell-free extracts and whole cells. Cells incubated with benzoate showed higher activation levels of phenol hydroxylase 1 (PH1) and PH3 (3.8 and 4.7 respectively), while the activation level of PH2 was significantly lower (1.7).

Analysis of cell germination after dormancy with phenol transcripts showed that the gene transcription for BDO was lower compared to cells treated with benzoate. The activation of 1,2-CDO genes with phenol induction was increased by 1.5 orders of magnitude and 5 times higher compared to induction by benzoate. From primers to known sequences of catechol 1,2-dioxygenase, the highest level of induction was observed with a pair specific to 1CP_849 bp_catA2_gb|FM877593.1| (catDO4) and 1CP_843 bp_*catA*_gb|X99622.2| (catDO6) and the level of expression of the first enzyme of the above was lower. *cat*A is part of the previously cloned operon, which was thought to be involved in the degradation of benzoate by this strain [49].

It should be noted that all phenol hydroxylases whose primers were synthesized using known sequences, were induced in the 1CP cells. However, there was a significant difference in the transcription levels of these enzymes. Gene activation for PH1 and PH2 was 2 orders of magnitude while for PH3 it was nearly 2000 times higher. The results of measurement of the total activity of PH, determined by the level of oxygen consumption by whole cells, indicate similarity with the published substrate profile for oxygenase component PheA1 of PH3 from the same strain (PheA1(c)), induced by phenol [50]. The activity of this PH with 2CP was comparable to that of phenol [50]. Unfortunately, the lack of data on the activity of PH with 4-CP and 2,4-DCP did not allow further comparison.

The issue of gene induction patterns in bacterial cell grown on phenol and benzoate has been discussed in several papers. Previous research does not definitively answer which enzyme is induced in strains grown on benzoate: the versatile BDO enzyme or potentially other initial attack enzymes. Mazzoli et al. (2007) proposed that benzoate induces the expression of two operons for degradation of benzoate and phenol [51]. An argument in favor of this assumption includes the rapid adaptation of cells pre-exposed to benzoate to phenol [51]. Molecular data obtained by the authors convincingly showed that benzoate induced the synthesis of the entire range of enzymes involved in the conversion of this compound to enol lactone. However, the oxygenase component of phenol hydroxylase was not found in cells grown on benzoate [51]. It is possible that the BDO enzyme in this strain could be responsible for the initial attack on phenol. Thus, the substrate specificity of enzymes involved in the degradation of phenol by strain *Acinetobacter radioresistens* S13 remains a topic of uncertainty [51].

The proteomic analysis of a number of bacterial strains was performed. Li et al. (2016) has conducted comparative proteomic analysis of phenol degradation process by *Arthrobacter* strain isolated from effluent in China [45]. The proteomic analysis of *Acinetobacter* strain Y cells grown on non-phenolic substrate showed high background expression of catechol 1,2-dioxygenase activity [6], which might help in the degradation of phenol at high (1 g L^−1^) concentrations. The qPCR data revealed approximately constant level of Cat 1,2-DO activity expression throughout the process of phenol destruction [6].

A gene cluster *catRBCA*, involved in catechol degradation and encoding *ortho*-pathway genes for catabolic metabolism of phenol, was isolated from a moderately halophilic bacterium *Halomonas organivorans* G-16.1 (CECT 5995(T)) [48]. The genes *catA*, *catB*, *catC* and the divergently transcribed *catR* code for 1,2-CDO, *cis*,*cis*-muconate cycloisomerase, muconolactone *delta*-isomerase and a LysR-type transcriptional regulator, respectively. The expression of *cat* and *ben* genes by phenol and benzoic acid was shown via RT-PCR analysis to determine whether the degradation genes are specifically induced and expressed in the presence of phenol and benzoate [48]. *H. organivorans* was grown in saline minimal medium using phenol and benzoate as the sole carbon source. Results of RT-PCR using the primer sets *catB*-*catC* and *catC*-*catA* showed that the *cat* genes are induced by phenol and benzoate, and they are co-transcribed in one single operon [48]. Results obtained with the primer set *benA*-*benB* demonstrated the induction of *ben* genes in the presence of phenol and benzoate, showing the co-transcription of both genes (*benAB*) [48]. However, no signals were reported to be obtained with cells grown in the presence of glucose [48].

### 4.3. Genetic Rearrangement in Cells after Long Storage

The chromosomes of microorganisms are complex and dynamic, which gives flexibility to the genome of the host strain [52]. Genomic instability may result from point mutations or rearrangements due to deletions, duplications, insertions, inversions, or translocations. Most forms of genome rearrangement result in the appearance of new sequences, structural impacts to the chromosome as a whole, and can have an indirect effect on the phenotype [53]. Traditionally, genomic rearrangements are detected by comparing complete sequenced genomes of microorganisms. However, this requires additional costs and may not always be cost-effective, especially when studying the influence of different cultivation conditions of one particular strain on its genome rearrangements. Therefore, preliminary biomolecular analysis can help in deciding the feasibility of further whole-genome sequencing. PCR fingerprinting methods rely on the electrophoretic separation and subsequent visualization of a set of amplified DNA fragments of varying sizes, thereby creating species-specific or strain-specific genomic fingerprint patterns (bands). These stripes (fingerprints) can be analyzed using computer programs and used to construct phylogenetic trees [54].

One of the fingerprinting methods based on the analysis of the entire bacterial genome is rep-PCR (repetitive sequence-based PCR). The essence of the rep-PCR method is the amplification of highly conserved regions between repeated DNA sequences randomly located throughout the bacterial genome [38]. There are four types of conserved repeat sequences used in rep-PCR based genomic fingerprinting: repeat extragenic palindromic sequences (REP), enterobacterial repeat intergenic consensus sequences (ERIC), BOX sequences and polytrinucleotide sequences (GTG)5 [38]. The PCR-based fingerprinting method is currently widely used for the molecular differentiation of microorganism strains not only belonging to the same genus, but also to the same species, the latter option being used most often [55,56,57,58]. Nevertheless, based on the data obtained, we can conclude that some rearrangements occur in the genome of the strain under study, although not significant, since the rep-PCR results do not allow us to identify significant differences in the samples. It can be assumed that in the genome of a bacterial culture, without the influence of external factors, some of the cells of the population undergo reversible genome rearrangement. Such rearrangements contribute to the survival of the bacterial culture when exposed to unfavorable/new environmental conditions. Some cells have one rearrangement, and some have another; some cells in the population do not undergo rearrangement and, thanks to such genomic plasticity, a microorganism can adapt change in environmental conditions for some reasons. Starvation and a long period of dormancy, up to 4 months, followed by germination in a mineral medium with phenol, can cause these minor changes. However, this issue requires further research.

### 4.4. Monitoring Phenol Degradation by Immobilized versus Free Cells

Immobilization has a positive effect on the process of microbial destruction of toxic compounds. Cells of *R. opacus* 1CP, immobilized on fiber, were able to decompose up to 1.0 g L^−1^ phenol. These data are comparable with the data on the decomposition of 1.0 g L^−1^ phenol by strain *P. aeruginosa* MTCC 4997 isolated from effluents collected from petrochemical industries near Mumbai, India [3]. Culture *P. putida* F1 were not able to degrade phenol in the concentration of 1.0 g L^−1^, degradation of 500 mg L^−1^ of phenol was completed in 5 days [59]. Unidentified bacterial mixture was able to degrade not only phenol, but also 2,4,6-trichlorophenol and pentachlorophenol [60]. This process was more rapid and the concentration of degraded phenol was higher at co-metabolic conditions, in the presence of glucose. Cells of *Chlamydomonas reinhardtii* immobilized in alginate removed up to 1300 μmol L^−1^ phenol during 10 days of cultivation [61].

The positive influence of starvation on degradation activity was shown earlier in respect of this culture, R. opacus 1CP [28] as well as *P. putida* P300 degrading chlorophenols [62]. Cells of *P. putida*, immobilized in polyvinyl alcohol gel pellets in a bubble column bioreactor showed higher growth and degradation rates against 2,4-dichlorophenol after starvation compared to vegetative cells [62]. Low carbon content was shown to enhance the arsenic oxidation ability of the strains across different genera in *Proteobacteria* [63]. A strong co-relation between carbon starvation and arsenic oxidation ability [63], and the positive influence of starvation on the degradation activity of a number of bacteria [28,62,64] provide an important basis for designing effective biopreparations for bioremediation of contaminated environments. 

## 5. Conclusions

In this study, an investigation was conducted regarding *R. opacus* 1CP actinobacteria cell viability under conditions of carbon source depletion. The subsequent results showed high viability rate, the ability to form dormant cells under prolonged starvation, and the capability to degrade phenol, a toxic compound. The formation of dormant cells is the main mechanism of overcoming unfavorable conditions by non-sporulating bacteria. An exit from the resting state following carbon source restoration leads to a vegetative growth of the culture. One peculiarity of germinating cells is their high metabolic activity. Increasing expression levels of genes encoding the enzymes of different degradation pathways were found in germinating cells. This explains the rapid adaptation of cells to the decomposition of degraded substrates after the dormant stage. Following dormancy, cells are capable of effectively destroying toxic compounds, regardless of the initial growth substrate. This opens up prospects for the use of any organic waste that can be used as a carbon source for cell growth, and for the production of microbial biomass. Direct immobilization of dormant cells on carriers allows for the stage of cell growth and induction before immobilization to be omitted. Good preservation of biodegradable activity in preparations of immobilized cells facilitates their use in the elimination of local contaminants with target toxicants.

## Figures and Tables

**Table 1:** Primers used for RT-PCR.

Gene	Forward Primer	Reverse Primer
Benzoate 1,2-dioxygenase	TACCACGTGTCCGCCACCC	CGTCGCGACGCTCGTTCAG
Catechol 1,2-dioxygenase 2	CACGCGCACGGGACAAATG	GCGCCATTCAACCCGTCG
Catechol 1,2-dioxygenase 3	CCCCTTTTTCGTCGCCGAC	CGTGCGGAATCGGGTATGG
Catechol 1,2-dioxygenase 4	CGCACCTACCGGAATGGAAC	CCGGCGAAGTACAGTTGGGT
Catechol 1,2-dioxygenase 6	GCAGTGGCTCATCGACGTG	ACCTGGCCGGAGAAGACG
Phenol hydroxylase 1 small subunit	TGACCTACGGGTGGATGGG	ACGATGAGGCCCGAGTCG
Phenol hydroxylase 2 small subunit	CCCGCGGATCAAAGAGATCA	CGCGGACGTACTTGTCGAGG
Phenol hydroxylase 3 small subunit	GCCTCTACGACGCGATGCAC	ATCGTCGGAGTTCTTCGGCG
*Beta*-ketoadipate enol-lactone hydrolase	TCGTCCGTTCGACCTCGACG	CGACATTCCCAGGACGTGCG
Protocatechuate 3,4-dioxygenase *alpha* subunit	ACCCGGTCTTCGCCAAGAGC	GACACGTCGATTCGTCCCGG
Chlorocatechol 1,2-dioxygenase	CATGATCAGCGTCGGCGAGG	GAACGGTCCTTGGATCGCACTG
Muconolactone *delta*-isomerase	AGGCCGAGGGCAAGATCGTG	TCACCGGCGTGACCTCAACG
RNA polymerase *beta* subunit	GTGTACTCCTCGCCTGCCG	GATCGTCGCCTGACGCTTC
16S RNA	ATGCAAGTCGAGCGGTAAG	ATGCAGCCGAAGGTCATATC

**Table 2:** Gene activation in germinating after dormancy *R. opacus* 1CP cells grown with benzoate or phenol as carbon source.

Genes Coding Following Enzyme	Growth Substrate		Gene’s Location *
	Benzoate *	Phenol *	
Benzoate 1,2-dioxygenase	263.60 ± 34.11	6.04 ± 1.83	chr
Catechol 1,2-dioxygenase 2	1.44 ± 0.61	1.47 ± 0.26	pR1CP1
Catechol 1,2-dioxygenase 3	3.07 ± 0.92	1.10 ± 0.17	chr
Catechol 1,2-dioxygenase 4	1.29 ± 0.12	2.57 ± 0.36	chr
Catechol 1,2-dioxygenase 6	29.65 ± 5.13	157.07 ± 20.22	chr
Phenol hydroxylase 1	3.84 ± 0.86	301.34 ± 33.18	pR1CP1
Phenol hydroxylase 2	1.71 ± 0.21	216.05 ± 10.19	chr
Phenol hydroxylase 3	4.72 ± 0.95	1917.80 ± 98.19	chr
*Beta*-ketoadipate enol-lactone hydrolase	1.73 ± 0.31	1.43 ± 0.23	chr
Protocatechuate 3,4-dioxygenase	23.75 ± 3.16	88.35 ± 14.16	chr
Chlorocatechol 1,2-dioxygenase	1.21 ± 0.54	1.24 ± 0.25	pR1CP1
Muconolactone *delta*-isomerase	1.04 ± 0.15	1.56 ± 0.61	chr

* The numbers represent benzoate cells/dormant cells and phenol cells/dormant cells ratios. pR1CP1 and chr stand for the pR1CP1 megaplasmid and the *R. opacus* 1CP chromosome.

**Table 3:** Enzyme activity (U (min × mg of protein)^−1^) in crude extract of *R. opacus* 1CP cells grown after dormancy in mineral medium with benzoate/phenol as the sole carbon source.

Enzymes	Growth Substrate	
	Benzoate	Phenol
Catechol 1,2-dioxygenase	0.2224 ± 0.0151	0.0288 ± 0.0011
Muconate cycloisomerase	0.0175 ± 0.0023	0.0565 ± 0.0017
Catechol 2,3-dioxygenase	0	0
Protocatechuate 2,3-dioxygenase	0	0
Protocatechuate 3,4-dioxygenase	0.0935 ± 0.0037	0.195 ± 0.009
Protocatechuate 4,5-dioxygenase	0	0
Gentisate 1,2-dioxygenase	0	0
Phenol hydroxylase	0	0
Salicylate hydroxylase	0	0

## Data Availability

Data are contained within the article.

## Abbreviations

BA	benzoate
BDO	benzoate 1,2-dioxygenase
Cat	catechol
1,2-CDO	catechol 1,2-dioxygenase
CLC	cyst-like cell
2-CP	2-chlorophenol
3-CP	3-chlorophenol
4-CP	4-chlorophenol
2,4-DCP	2,4-dichlorophenol
LB medium	Luria-Bertani medium
MLI	muconolactone isomerase
2,4,6-TCP	2,4,6-trichlorophenol
PCA	protocatechuate
PCA 3,4-PCDO	protocatechuate 3,4-dioxygenase
PH	phenol hydroxylase
